# Pulmonary Innate Immune Response and Melatonin Receptors in the Perinatal Stress

**DOI:** 10.1155/2013/340959

**Published:** 2013-01-16

**Authors:** Janaínna Grazielle Pacheco Olegário, Marcos Vinícius Silva, Juliana Reis Machado, Laura Penna Rocha, Marlene Antônia Reis, Camila Souza de Oliveira Guimarães, Rosana Rosa Miranda Corrêa

**Affiliations:** ^1^Discipline of General Pathology, Triângulo Mineiro Federal University, Avenida Frei Paulino No. 30, Bairro Abadia, 38025-180 Uberaba, MG, Brazil; ^2^Immunology Department, Triângulo Mineiro Federal University, Avenida Frei Paulino No. 30, Bairro Abadia, 38025-180 Uberaba, MG, Brazil

## Abstract

*Objective*. To analyze the cytokines of the innate immune pulmonary response and the capacity for local response to melatonin according to the perinatal stress. *Methods*. 49 cases of pediatric autopsies were evaluated, divided according to cause of death, perinatal stress, gestational age, and birth weight. The percentages of IL-6, C-reactive protein (CRP), IL-1**β**, TNF-**α**, and melatonin receptor were evaluated by immunohistochemistry. *Results*. The IL-6 expression was higher in the children showing chronic stress, anoxia, and infection. The IL-6 expression showed a progressive increase according to the relation between weight and GA. There was no significant difference in the expression of IL-1**β** and TNF-**α**. The CRP expression was higher in the cases showing chronic stress and premature cases. The expression of melatonin receptors was significantly higher in the cases showing chronic stress, being more evident in the cases showing infection. *Conclusion*. The cause of death and the type of stress influence the expression *in situ* of melatonin and cytokines of the innate immune pulmonary response. The evaluation of IL-6 and CRP may contribute to the understanding of the evolution of neonates with chronic stress. The greater sensitivity of the lung to melatonin in these cases may indicate an attempt at controlling the immunological response, in an attempt to diminish the harmful effects of stress.

## 1. Introduction

Gestational complications can stimulate the liberation of soluble factors involved in the installation of the “state of stress” [1, 2]. Diagnosis and the possible effects of lesions in the perinatal period frequently suffer interference from the physiological conditions of the child, modifying the morphological characteristics of some organs which are called “stress organs.” Among the most important ones are the adrenal, the liver, the lungs, and the thymus [3]. 

The fetuses with stress show endocrinal alterations, expressing an abnormal ratio for cortisol/dehydroepiandrosterone [2] and activation of the innate fetus immune system, running with an increase of IL-6 and of other cytokines in the plasma, such as IL-1*β*, TNF-*α* [4, 5]. It is related to other serious cases of neonatal morbidity, such as respiratory distress syndrome, pneumonia, bronchopulmonary dysplasia, necrotizing enterocolitis, cerebral palsy, sepsis, and death [6, 7].

Melatonin is an important regulator of the circadian rhythm and has antioxidizing, anti-inflammatory, and immunomodulating properties. It is an indoleamine, and these have been used in the treatment of neonatal sepsis and bronchopulmonary dysplasia. In sepsis, melatonin avoids the recruitment of leukocytes to the affected organs, balancing the inflammation [8, 9]. It was shown that melatonin lowers interleukin IL-6, IL-8, TNF-*α*, and nitrite/nitrate levels and modifies serum inflammatory parameters in surgical neonates, thus improving their clinical course [10]. In newborns with sepsis, distress, or other conditions where there are excessive reactive oxygen and nitrogen species production, it is reported that melatonin reduces the oxidative stress [11] and the levels of nitrite/nitrate and lipid peroxidation products [12], suggesting that melatonin may be an effective protective agent for the fetus [13]. 

Thus, understanding the working mechanisms of the cytokines of the innate immune pulmonary response in the pulmonary morphology, as well as the capacity for local response of this organ by means of the protective substances such as melatonin, is important step in the understanding of the evolution of pulmonary illnesses. Thus, the purpose of this study was to analyze the expression of the innate immune pulmonary response markers and the pulmonary local response by means of melatonin, according to the causes of the perinatal death.

## 2. Materials and Methods

This study was approved by the Triângulo Mineiro Federal University (UFTM) Research Ethics Committee, approval number 1316. 

Lung fragments of perinatal autopsies were conducted from the 22nd gestational week to the 7th postnatal day in the General Hospital of Triângulo Mineiro Federal University, Minas Gerais, Brazil. We excluded the following cases: perinatal autopsies with incomplete records and protocols, blocks and slides not available in the archives; cases of autolyzed lungs; and live births submitted to ventilation, intubation, or surfactant replacement. A matching was performed according to gestational age (GA) and cause of death, and 49 cases were obtained.

The groups of the cause of death were defined according to [14] as congenital malformation, isoimmunization, antepartum asphyxia, intrapartum asphyxia, birth trauma, pulmonary immaturity, hyaline membrane disease, intracerebral hemorrhage, infection, miscellaneous, unclassified, or unknown. For the morphologic analysis, only the most frequent causes of death were included, such as congenital malformation, perinatal anoxia before delivery, and ascending infection; autopsies of other causes of death were excluded. 

The cases included in this study were classified according the perinatal stress. Perinatal stress was defined when the thymus, adrenal, and liver presented morphological alterations compatible with intrauterine stress. The adrenal presented increasing amounts of coarse lipid droplets in the fetal cortex. The thymus was evaluated according to the presence of phagocytosis (positive cells for CD68 antibody), cortex thickness, and the weight for involution. The amount of intrahepatic hematopoiesis was evaluated in the liver. The placenta was also examined in these cases in order to confirm the autopsy findings. The cases were subdivided into two groups: acute and chronic perinatal stress. The acute stress was characterized by events occurring at or after birth and might have been the causative agent of fetal death. On the other hand, chronic stress was defined by the response to injuries of long duration which begin in the intrauterine period and remain until birth and may be related to the pathogenesis of perinatal death [15, 16]. 

Data about anthropometric measurements, weight, GA, and clinical complications was collected from the autopsy records. GA was determined through hallux-calcaneus length [17]. Children with GA less than 37 weeks were considered premature [18] and those with GA greater than 20 weeks and Apgar's score zero in the first minute were considered stillborn [19]. The children were classified according to the relation between weight and GA in small for gestational age (SGA, weight is below the 10th percentile), appropriate for gestational age (AGA, the weight is between 10th and 90th percentile), and large for gestational age (LGA, weight is above the 90th percentile) [20].

Immunostaining was done as a single batch, by using the following primary antibodies: anti-IL-6 (1 : 600 Abcam), anti-IL-1*β* (1 : 100 Abcam), anti-TNF-*α* (1 : 50 DBS), anti-C reactive protein (CRP) (1 : 1000 Abcam), and anti-*melatonin receptor 1B* (1 : 100 Abcam).

Quantization of immunostained markers in the lung was performed using a video camera connected to a conventional light microscope and to a computer with Leica QWin Plus image analysis software. The cumulative average method was used to determine the number of measures [21], showing a pattern result of 60 measures per slide. The results were expressed in percentage of the immunostained area per field.

The statistical analysis was conducted with *SigmaStat 2.03* software. In cases of normal distribution and similar variances, Student's *t*-test was used in the comparison of two groups. Otherwise, Mann-Whitney (*T*) was used in the comparison between two groups and Kruskal-Wallis (*H*) or ANOVA (*F*) in the comparison between three or more groups. Correlation between the two variables with non-normal distribution was analyzed by the Spearman rank correlation test Spearman (Sr). Differences in which “*P*” was less than 5%  (*P* < 0.05) were considered statistically significant.

## 3. Results

Among the 49 cases of autopsied children in the perinatal period, 20 (40.81%) were diagnosed with perinatal anoxia, 14 (28.57%) with ascending infection, and 15 (30.62%) with malformations. The average gestational age was 3.17 ± 5.76 in perinatal anoxia, 31.14 ± 6.41 in ascending infection, and 31.93 ± 6.04 in malformations. We evaluated 28 stillbirths, with mean of gestational age of 30.12 weeks. The live births presented an average of 10 hours of postnatal life.

The IL-6 expression was higher in the children showing chronic stress, anoxia, and infection (*P* ≤ 0.001). The IL-6 expression showed a progressive increase according to the relation between weight and GA, with values of 14.55 ± 10.14 in SGA, 19.73 ± 10.56 in AGA, and 25.62 ± 12.29 in LGA. There was no significant difference in the expression of IL-1*β* and TNF-*α* (Table 1). The CRP expression was higher in the cases showing chronic stress (*P* ≤ 0.001), being more accentuated in the cases with infection, and followed by the cases with anoxia (*P* ≤ 0.001). There was an increased expression of this mediator in the premature cases (Table 1).

The expression of melatonin receptors was significantly higher in the cases showing chronic stress (*P* = 0.028), being more evident in the cases showing infection (Table 1).

**Figure 1:**
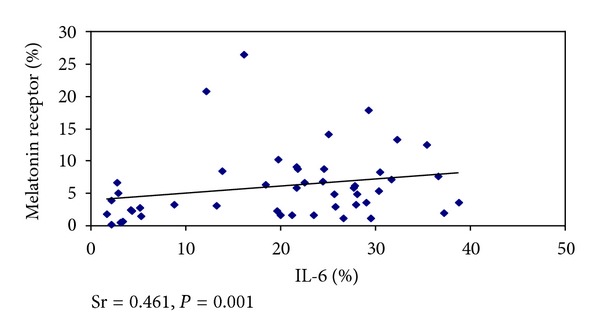
Correlation between IL-6 and melatonin receptors.

**Figure 2:**
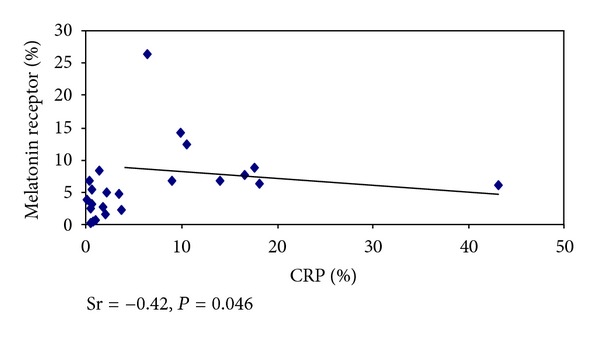
Correlation between C-reactive protein (CRP) and melatonin receptors.

There was a positive and significant correlation between IL-6 and melatonin receptors (Sr = 0.461, *P* = 0.001) (Figure 1) and negative and significant one when the CRP values and the melatonin receptors were assessed (Sr = − 0.420, *P* = 0.046) (Figure 2). There was no significant correlation between IL-6 and CRP.

## 4. Discussion

In our data, IL-6 was increased in the cases with chronic stress, in which there is a longer exposure period to the stimulus. A study concerning the immune response in newly born babies showed that the IL-6 shows a peak of production 2-3 hours after the stimulus for its production [22]. As all our cases went on to death, we believe that the stimulus which causes lesions could have provoked a higher immunological imbalance than that commonly described in live, and due to this, a production of higher levels of IL-6 after a longer period of exposure to the initial stimulus.

The IL-6 expression showed a progressive increase according to relation between weight and GA. Our cases show that, even with an established inflammatory picture, there is an increase of the body weight and not a decrease, as expected [23]. The increase of cytokines released during the inflammatory process could also contribute to the edema, particularly IL-6 and TNF-*α*, which favor the production of glucocorticoids and other steroids by the adrenal cortex [24]. These steroids increase the concentration of the free fatty acids and glycerol [25], inhibit lipolysis, and favor lipid accumulation [26]. Therefore, the weight as fetal prognosis factor must be carefully evaluated, since the intensity of the stress factor may interfere directly in this parameter. Our data demonstrate that, under these conditions, IL-6 may be a good marker for stress, demonstrating the fetal state more accurately than the weight.

There was no significant difference in the expression of IL-1*β* and TNF-*α*. One of the first cytokines in the inflammatory process is TNF-*α*, which is produced locally and is considered a proinflammatory cytokine. In conjunction with IL-1*β*, TNF-*α* stimulates the production of IL-6 [27]. Studies that use the model of ischemia and the reperfusion, promoting acute stress, relate a rise in TNF-*α* to the inflammatory stimulus associated to the early tissue damage [28]. However, a study shows that there is no variation in the TNF-*α*, days after installation of neonatal sepsis [29]. We believe that the stimuli for production of these mediators are varied, being considered an acute mediator more by the fact of its first appear, but these are still present in the continuous stress. 

The CRP expression was higher in the cases with chronic stress, mainly in the cases with infection. CRP is considered a good marker for infection [30] and is found in higher quantity in cases with chorioamnionitis [31] and neonatal sepsis [32], being more specific for the later stages [33]. This data is in accordance with our results, which show a rise in the CRP expression in the cases of chronic stress, showing that the length of time exposure is important so that the detection of the CRP be sufficient for the diagnosis of intrauterine alterations. The higher CRP expression in the cases of anoxia indicates that this marker must also be verified when the tissue lesion occurs due to oxygen privation and not strictly related to the presence of an infectious agent.

The amount of CRP was higher in the premature cases. The premature fetuses may respond in a very intense manner when faced with intrauterine alterations, and this response may start off a premature birth, in a fetal attempt to avoid the hostile environment [34]. Thus, apart from the stimulus by means of anoxia or infection, the amount of CRP rises in different degrees, being more accentuated in children that do not reach gestational maturity, probably due to the higher degree of stress to which they are submitted.

The percentage of the melatonin receptors was higher in the groups with chronic stress and with the cause of death associated to infectious cases. Previous studies report an increase in the receptors for melanin and of enzymes involved in its synthesis in the placentas of children that suffered intrauterine stress, independent of the cause [16]. Melatonin has an anti-inflammatory effect, reducing the expression of the nuclear factor kappa B, and with this, there is the production of proinflammatory cytokines and chemokines, decreasing the leukocyte adhesion to the endothelium, the recruitment of neutrophils, the synthesis of nitric oxide in the septic shock, and the enzymes associated to the oxidative stress [8, 9, 35]. In this manner, its presence in higher amount in the cases of infection may mean an attempt to assuage the damage caused by serious infections that result in cases of intense inflammatory response. 

There was a positive and significant correlation between the values for IL-6 and melatonin receptors and negative and significant one when the values for CRP were assessed along with the melatonin receptors. Due to the fact that they show anti-inflammatory functions, its rise in association to the rise in IL-6 may represent a control response from the immune response. IL-6 is the stimulus to CRP production, and CRP is considered a good marker for infection in later stages [33, 35]. In our data, CRP was higher in chronic stress and infection, and there was no significant correlation between IL-6 and CRP. All our cases died, so we believe that CRP levels observed may represent extreme values for its expression. Thus, in cases of chronic stress, IL-6 is first increased, and the body tries to control the immune system by increasing the melatonin receptor. When stress is extended and becomes severe, the levels of CRP rise in response to IL-6, but due to gravity the organism loses its ability to respond to injury, in our data demonstrated by reduced expression of melatonin receptors.

## 5. Conclusion

The cause of death and the type of stress influence the expression *in situ* of melatonin and cytokines of the innate immune pulmonary response. The evaluation of IL-6 and CRP may contribute to the understanding of the evolution of neonates with chronic stress. The greater sensitivity of the lung to melatonin in these cases may indicate an attempt of control of the immunological response, in an attempt to diminish the harmful effects of stress. 

## Figures and Tables

**Table 1:** Comparison between the amounts of inflammatory markers and melatonin receptor in the lung.

Groups	*N* (%)	IL-6	IL1-*β*	TNF-*α*	CRP	Mel Rec
		*X* ± SD/median (minimum–maximum)				

Chronic stress	32 (63.31)	25.37 (13.20–19.65)	1.42 (0.16–6.38)	2.90 (0.10–10.55)	9.45 (0.65–43.05)	5.95 (1.08–26.36)
Acute stress	17 (36.69)	4.30 (1.65–26.61)	1.19 (0.27–5.98)	3.59 (0.97–9.47)	0.63 (0.09–2.17)	2.79 (0.21–20.82)
		*T* = 212.000; *P* ≤ 0.001	*T* = 340.000; *P* = 0.809	*T* = 328.000; *P* = 0.691	*T* = 52.000; *P* ≤ 0.001	*T* = 308.000; *P* = 0.028

Term	12 (24.49)	20.37 ± 14.00	1.12 (0.43–4.97)	3.59 ± 2.79	1.48 (0.34–16.62)	5.21 ± 3.29
Preterm	37 (75.51)	19.66 ± 9.98	1.42 (0.16–6.38)	3.96 ± 2.58	3.45 (0.09–43.05)	6.33 ± 5.99
		*t* = 0.194; *P* = 0.847	*T* = 245.000; *P* = 0.945	*t* = 0.411; *P* = 0.683	*T* = 61.000; *P* = 0.462	*t* = 0.614; *P* = 0.543

Anoxia	20 (40.82)	25.51 ± 6.22*	1.59 (0.16–6.38)	2.99 (0.19–9.44)	3.62 (0.65–43.05)*	4.89 (1.08–17.84)
Infection	14 (28.57)	25.07 ± 7.45^#^	1.33 (0.38–4.68)	2.83 (0.49–10.55)	10.19 (6.47–18.16)^#^	7.58 (1.62–26.36)^#^
Malformation	15 (30.61)	7.36 ± 8.13^∗#^	1.06 (0.27–5.98)	2.97 (0.97–9.47)	0.63 (0.09–2.17)^∗#^	2.51 (0.21–20.82)^#^
		*F* = 32.483; *P* < 0.001	*H* = 0.251; *P* = 0.882	*H* = 0.924; *P* = 0.630	*H* = 13.357; *P* = 0.001	*H* = 12.489; *P* = 0.002

CRP: C-reactive protein; Mel Rec: melatonin receptor. *X* ± SD: mean ± standard deviation; ^∗#^significant difference between groups by multiple comparison tests.
